# Comparison of the Fruit Volatile Profiles of Five Muscadine Grape Cultivars (*Vitis rotundifolia* Michx.) Using HS-SPME-GC/MS Combined With Multivariate Statistical Analysis

**DOI:** 10.3389/fpls.2021.728891

**Published:** 2021-10-25

**Authors:** Honghong Deng, Runmei He, Meicun Long, Yanmei Li, Yuanyuan Zheng, Lijin Lin, Dong Liang, Xiaoai Zhang, Ming'an Liao, Xiulan Lv, Qunxian Deng, Hui Xia

**Affiliations:** Institute of Pomology and Olericulture, College of Horticulture, Sichuan Agricultural University, Chengdu, China

**Keywords:** volatile organic compounds, headspace solid-phase microextraction, gas chromatography-mass spectrometry, principal component analysis, partial least-squares discriminate analysis

## Abstract

Fruit aromas are composed of a complex mixture of volatile organic compounds, which are essential attributes associated with the overall flavor and consumer preference. Muscadine grape (MG; *Vitis rotundifolia* Michx.) is an aroma-dense fruit crop. However, there is very scarce information on its volatile profiles. In this study, the volatile constituents of five newly introduced MG cultivars, including Alachua, Carlos, Fry, Granny Val, and Noble, were profiled using headspace solid-phase microextraction-gas chromatography-mass spectrometry (HS-SPME-GC/MS) combined with multivariate statistical analysis. A total of 44 compounds, including esters, aldehydes, alcohols, fatty acids, terpenes, ketones, and furan, were identified and relatively quantified. Principal component analysis (PCA) and partial least-squares discriminant analysis (PLS-DA) evidently discriminated against the five MG cultivars based on their volatile profiles. The specific volatiles that contributed the most to this discrimination were highlighted. Geraniol and cinnamyl alcohol were demonstrated to be essential for characterizing the Alachua MG cultivar, whereas ethyl trans-2-butenoate and propyl acetate were shown to be important compounds to characterize the Noble MG cultivar. The results further showed that 2-Ethyl-1-hexanol, (*Z*)-3-hexenal, and (*E*)-2-hexenol were closely related to Carlos, Fry, and Granny Val cultivars, respectively. This investigation is the first in-depth exploration of the volatile profiles of the aroma-dense muscadine grape, which is essential for future genetic or biotechnological improvements to attain a cultivar with the desired flavor.

## Introduction

Grapes (*Vitis vinifera* L.) are among the most economically important and the earliest domesticated fruit crops throughout the world (Reisch et al., 2012; Ramos-Madrigal et al., 2019). Similar to fresh fruit, grapes are an essential nutrient-dense food source, being part of the human diet, while as processed products, i.e., dried raisins, fruit preserves, primarily wine, and spirits, they have added economic values and represent a globally traded commodity with significant economic value (Reisch et al., 2012; Ramos-Madrigal et al., 2019). Most of the grapes cultivated for fruit production are either of the species *V. vinifera*, hybrids of *V. vinifera*, belonging to the genus *Vitis* L., or, to a lower extent, to the closely related subgenus *Muscadinia* (Reisch et al., 2012; Lin et al., 2019).

Taxonomically, the muscadine grape (MG) (*Muscadinia rotundifolia* Michx., syn. *V. rotundifolia* Michx.) is closely related to *Vitis* species (Olien, 1990; Liu et al., 2016; Wen et al., 2018). Genetically, the basic chromosome of MG (x = 20) is interestingly one chromosome greater than the 19 basic chromosomes of the *V. vinifera* L. (Olien, 1990). Indigenous to the warm and humid climate of the southeastern United States, MG was the first American grape species to be cultivated (Olien, 1990) and has been cultivated for more than 400 years (Stanley, 1997; Conner, 2009). It can be found growing naturally from Delaware to Central Florida state, i.e., south-north distribution, and from Texas and Oklahoma to the eastern coastal plain, i.e., east-west span, in the United States, along the Mississippi River to Missouri and near the Appalachian Mountains from the east and west (Olien, 1990).

Muscadine grape has relatively high intrinsic resistance to disease, including downy and powdery mildews, gray mold, and anthracnose, especially Pierce's disease (*Xylella fasti diosa*) (Olien, 1990). Over the past few decades, MG has drawn increasing attention from consumers, growers, and breeders because it constitutes an important source of dietary fibers, amino acids, minerals, vitamins, antioxidant phytochemicals, such as polyphenolic compounds, and other health-promoting compounds (Sandhu and Gu, 2010; Wang et al., 2010). Since the resurgence of the interest in MG in the United States to date, more than 100 MG cultivars have been released (Hoffmann et al., 2020), and MG has also been extended to California, Chile, and China (Hoffmann et al., 2020). For example, five MG cultivars, including Alachua, Carlos, Fry, Granny Val, and Noble, have been successfully introduced and cultivated in southern China (Wei et al., 2017; Ren et al., 2020).

In addition to having significantly more phytonutrients than the average table grape cultivars (Marshall et al., 2012), MG has a distinct musky aroma and unique flavor (Kambiranda et al., 2016), which is essential for its current wine, juice, and table grape markets. Volatile organic compounds (VOCs), such as esters, ketones, terpenes, aldehydes, alcohols, C_13_ norisoprenoids, and benzenoids, are significant determinants of the grape aroma and perceived flavor (Gürbüz et al., 2014; Lin et al., 2019). The VOCs of fruits are complex and vary significantly depending on the analytical methods used and a plethora of factors, such as the cultivar, sample type, ripening stage, environmental conditions, abiotic and biotic stress (Sánchez-Palomo et al., 2005; Kalua and Boss, 2009; Wu et al., 2020). VOCs are the main contributors to the fresh and fruity note of wines (Perez-Coello and Diaz-Maroto, 2009) and are important nutritional constituents, shaping the sensory properties of foods and influencing the perception of consumers (Berger, 2009). Each fruit species has a specific combination of various VOCs with different concentration and perception thresholds (El Hadi et al., 2013). The differences of VOCs between the table and wine grapes have been well investigated, whereas only a few studies have reported the VOC compositions of MG (Lee et al., 2016).

Headspace solid-phase microextraction (HS-SPME) is a passive sampling approach that collects compounds present in the vapor phase above samples, which does not interfere with the samples and omits the compounds in the vapor phase above the matrix during heating (Lubes and Goodarzi, 2017). Compared with solvent extraction, HS-SPME is rapid, simple, and reproducible, and yields more realistic VOC discrimination results (Lubes and Goodarzi, 2017). HS-SPME coupled with gas chromatography-mass spectrometry (GC-MS) has been extensively employed to study the volatile profiles of many fruit varieties, vegetables, and beverages.

Muscadine grape is an aroma-dense fruit. However, relatively few publications have documented its aromatic VOC compositions. Therefore, a systematic, qualitative, and quantitative investigation of the MG VOCs is highly required and of great importance. In this study, HS-SPME-GC-MS was employed to compare, both qualitatively and quantitatively, the volatile constituents of five MG cultivars that were newly introduced in China. Multivariate statistical analysis methods, including principal component analysis (PCA), and partial least-squares discriminate analysis (PLS-DA), were utilized to highlight the differences among cultivars and identify the chemical biomarkers discriminating the five MG cultivars. Determining the VOCs of MG and exploring the differences in VOCs among cultivars is essential for future genetic or biotechnological improvements targeting aroma-dense grape cultivars.

## Materials and Methods

### Fruit Materials

This study was conducted using 5-year-old (in 2020) newly introduced MG cultivars (Alachua, Carlos, Fry, Granny Val, and Noble). Trees were grown in the fields of the experimental station of the College of Horticulture, Sichuan Agricultural University (30°42′N, 103°51′E), China. In this area, all vines were subjected to identical and standard viticultural practices for table grape cultivars throughout the experiment, including winter pruning, pest and pathogen control, basal fertilizer, and irrigation.

**Figure 1:**
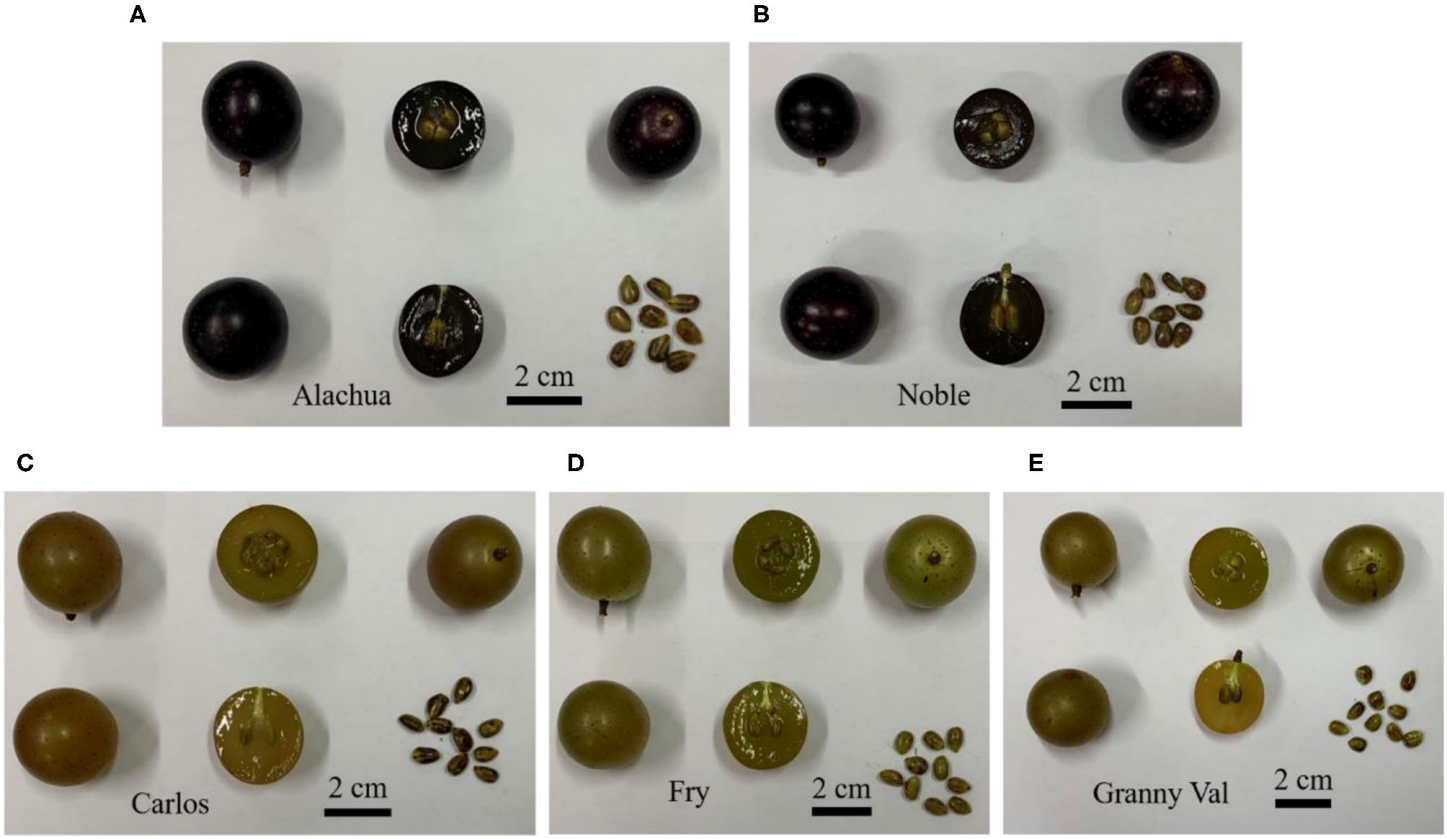
Fruit phenotypes of the Alachua **(A)**, Noble **(B)**, Carlos **(C)**, Fry **(D)**, and Granny Val **(E)** muscadine grape cultivars. Scale bars denote 2 cm.

For each cultivar, fruit samples (Figure 1) were randomly collected from three clusters of four different plants in September (the maturation stage), which is considered ideal for commercialization in Sichuan based on the observations during the previous years. Specifically, the solid soluble content of Alachua, Carlos, Fry, Granny Val, and Noble samples ranged from 16.28 to 17.7°Brix, from 14.2 to 16.18°Brix, from 14.62 to 16.3°Brix, from 14.9 to 18.03°Brix, and from 14.7 to 15.38°Brix, respectively. Each cluster was clipped off at the end of the peduncle. Samples were stored in a cold chamber and transported to the laboratory within 2 h. Three berries from the top, central, and bottom parts of each cluster were pooled together. A total of 36 berries were picked and randomly divided into three biological replicates. A 0.5 cm thickness slice was obtained from the equatorial region of each fruit, immediately frozen in liquid nitrogen, and stored at −80°C until the further determination of VOCs.

### Chemicals and Solvents

The study used *n*-Alkane (C_7_-C_30_) standards and the available authentic standards, including ethyl acetate (≥99.5%), propyl acetate (≥99.5%), butyl acetate (99.7%), ethyl trans-2-butenoate, ethyl hexanoate (≥99%), ethyl heptanoate (≥99%), ethyl octanoate (≥99%), ethyl 3-hydroxybutyrate (≥ 98%), hexyl hexanoate (≥ 98%), hexanal (≥95%), (*Z*)-3-hexenal (50% in triacetin), (*E*)-2-hexenal (≥97%), nonanal (≥99.5%), benzaldehyde (≥99.5%), (*E*)-2-decenal (≥95%), citral (≥95%), 1-butanol (≥99.4%), 1-hexanol (≥99.9%), (*E*)-2-hexenol (96%), 1-octen-3-ol (≥98%), 1-heptanol (≥99.5%), 2-ethylhexanol (≥99%), 1-octanol (≥99%), 2-octen-1-ol (97%), (*Z*)-5-octen-1-ol (≥97%), (*E*)-5-decen-1-ol (≥97%), phenylethyl alcohol (≥99%), cinnamyl alcohol (≥96%), acetic acid (≥99.7%), hexanoic acid (≥98%), heptanoic acid (≥99%), octanoic acid (99%), nonanoic acid (≥99.5%), limonene (mixture of D- and L-form at ratio of 1:1, ≥95%), linalool (≥99%), citronellol (≥95%), nerol (≥97%), geraniol (≥98.5%), 2-octanone (≥99.5%), acetophenone (≥99.5%), 2-pentylfuran (≥97%), 2-octanol (≥97%), and sodium chloride (NaCl, ≥99%) which were all purchased from Sigma-Aldrich (St. Louis, MO, USA). Geranic acid (sum of isomers, 98%) was purchased from Alfa Aesar Corporation (Tianjin, China). Ultrapure water was prepared using a Milli-Q water purification system (Millipore Corporation, Bedford, MA, USA) with a 0.22 μm filter.

### Fruit Sample Preparation for HS-SPME

The VOCs from the whole fruit were extracted using HS-SPME. Fruit samples, including the seeds, flesh, and skins, were pooled together and fully ground into a fine powder in liquid nitrogen. For each extraction sample, 100 mg of powder, spiked with 10 μl of 2-octanol, was accurately weighed and transferred to a 20-ml glass sample container (Thermo Scientific, Bellefonte, PA, USA). Samples were overlaid with a 5 ml saturated sodium chloride (NaCl) solution to inhibit enzyme degradation. A tiny stirring bar was added to facilitate VOC release before the glass vial was capped. The homogenized samples were incubated for 15 min in a 60°C water bath with continuous agitation (125 rpm). Thereafter, the VOCs were collected using a 2 cm DVB/CAR/PMDS SPME fiber (50/30 μm, Supelco Inc., Bellefonte, PA, USA) by exposing the fiber to the headspace for another 30 min under the same conditions. The fibers were activated before sampling according to the instructions of the manufacturer. After this incubation step, the SPME fiber was inserted directly into the injection port of the GC system for thermal desorption (4 min at 250°C) in a splitless mode.

### GC-MS Operating Conditions

Volatile organic compounds were analyzed using an Agilent 7890 gas chromatography system equipped with a 5977B mass spectrometer (Agilent Technologies Inc., Santa Clara, CA, USA). Helium (99.999% purity) was used as the carrier gas with a front inlet purge flow rate of 3 ml min^−1^ and a constant gas flow rate through the column of 1 ml min^−1^. VOC separation was carried out using an Agilent DB-Wax (30m × 250 μm ×0.25 μm, Agilent Technologies Inc., Santa Clara, CA, USA) column. The oven temperature program was initiated at 40°C for 4 min, then ramped up to 245°C at a rate of 5 °C min^−1^, and held for 5 min. The transfer line, ion source, and quadrupole mass detector temperature values were set to 250, 230, and 150°C, respectively. Mass spectra in electron impact ionization (ME-EI) mode were recorded at ionization energy of 70 eV. Data acquisition was performed using the mass spectrometer scanning mode from m/z 20 to 500. The solvent delay time was 0 min.

### VOC Identification and Quantification

The row peak obtained from GC-MS was first processed using Chroma TOF 4.3X software (LECO Corporation, St Joseph, MI, USA). The parameters used for raw peak extraction, data baseline filtering and calibration of the baseline, peak alignment, deconvolution analysis, peak identification, integration, and spectrum match of the peak area were the same for all samples. VOCs were identified by matching the retention time in conjunction with the MS fragments with the data of previous studies using a similar chromatographic column and the built-in commercial MS databases, such as the NIST reference library (National Institute of Standards and Technology, Gaithersburg, MA, USA). The VOCs were checked against authentic standards when they were accessible. The VOC concentrations were obtained *via* peak normalization and semi-quantified to the internal reference standard in the same GC-MS run, which was added at the beginning of the VOC extraction step, as mentioned above. Correspondingly, the peak area of each VOC was converted into a relative concentration value for the following statistical analyses.

### Statistical Analyses

The normalized data were exported to R free software v. 3.2.3 (R Core Team, 2020) for statistical analysis. The means and SEs were determined for all the detected variables. Significant variances were validated using one-way ANOVA and Tukey's honestly significant difference (HSD) test (*P* < 0.05). The data were then imported into SIMCA software version 14.1 (Umetrics, Umea, Sweden) for multivariate statistical analyses. PCA was first conducted to visualize the main correlations in the whole data matrix, followed by PLS-DA to discriminate the MG varieties further. A permutation test (100 times) was applied to validate the PLS-DA results and to avoid overfitting (Saccenti, 2012). Afterward, the variable importance in projection (VIP) was used to define which VOCs significantly contribute to discriminate the five MG cultivars (Chong and Jun, 2005).

## Results and Discussion

### Characterization of the Fruit VOCs of the Five MG Cultivars

Headspace-SPME combined with GC-MS analysis and the comparison of mass spectra with the NIST17 library and the available authentic standards resulted in the identification of 44 metabolites in the five MG cultivars, whose chromatographic profiles are shown in Supplementary Figure 1. These metabolites include eight esters, ten aldehydes, twelve alcohols, six fatty acids, five terpenes, two ketones, and one furan based on their chemical nature (Table 1). We found that this chemical classification agreed with the major constituents for grape volatiles that have been reported so far (Lee et al., 2016; Mencarelli and Bellincontro, 2018; Lin et al., 2019; Wu et al., 2020; Golombek et al., 2021; Ju et al., 2021).

**Table 1:** The chemical compositions and relative concentrations of fruit volatiles of five muscadine grape cultivars.

**Compound names**	**RI**	**Alachua**	**Carlos**	**Fry**	**Granny Val**	**Noble**
**Esters**						
Ethyl Acetate	982	1.95 ± 0.38a	3.92 ± 0.67a	3.15 ± 0.95a	2.61 ± 0.51a	3.91 ± 1.56a
Propyl acetate	952	0.03 ± 0a	0.03 ± 0.01a	0.03 ± 0.01a	0.03 ± 0a	0.09 ± 0.04a
Butyl acetate	1,060	0.54 ± 0.12a	0.93 ± 0.35a	0.49 ± 0.18a	1.05 ± 0.27a	1.78 ± 0.7a
Ethyl trans-2-butenoate	1,158	2.55 ± 0.73ab	0.67 ± 0.46b	1.36 ± 0.19b	2.24 ± 0.8ab	4.3 ± 0.33a
Ethyl hexanoate	1,241	1.19 ± 0.29a	0.64 ± 0.06a	0.77 ± 0.14a	0.5 ± 0.02a	0.53 ± 0.11a
Ethyl heptanoate	1,331	0.05 ± 0.02a	0.04 ± 0a	0.04 ± 0.01a	0.03 ± 0.01a	0.02 ± 0a
Ethyl octanoate	1,412	0.24 ± 0.09ab	0.37 ± 0.09ab	0.55 ± 0.08a	0.15 ± 0.05b	0.05 ± 0.01b
Ethyl 3-hydroxybutyrate	1,482	0.16 ± 0.03c	0.74 ± 0.09b	0.79 ± 0.07b	1.31 ± 0.18a	0.21 ± 0.06c
Hexyl hexanoate	1,599	0.08 ± 0.05a	0.02 ± 0a	0.02 ± 0a	0.01 ± 0a	0.01 ± 0a
Subtotal		6.78 ± 0.24a	7.37 ± 2.65a	7.21 ± 2.45a	7.94 ± 1.19a	10.90 ± 4.57a
**Aldehydes**						
Hexanal	1,072	2.67 ± 0.3a	2.82 ± 0.67a	2.04 ± 0.62a	2.17 ± 0.24a	1.44 ± 0.09a
(Z)-3-Hexenal	1,124	0.1 ± 0.01a	0.14 ± 0.02a	0.12 ± 0.04a	0.07 ± 0.01a	0.04 ± 0a
(E)-2-Hexenal	1,215	6.4 ± 0.31abc	8.09 ± 1.21ab	9.19 ± 1.26a	4.87 ± 0.72bc	3.04 ± 0.33c
(Z)-2-Heptenal	1,319	0.15 ± 0.01a	0.1 ± 0.03ab	0.09 ± 0ab	0.09 ± 0.01ab	0.07 ± 0.02b
Nonanal	1,392	0.07 ± 0.01b	0.17 ± 0.02a	0.15 ± 0.01a	0.14 ± 0.01a	0.05 ± 0b
Benzaldehyde	1,520	0.17 ± 0.01bc	0.64 ± 0.15a	0.5 ± 0.08abc	0.52 ± 0.07ab	0.13 ± 0.03c
Benzeneacetaldehyde	1,630	1.07 ± 0.33a	2.14 ± 0.31a	1.8 ± 0.05a	2.39 ± 0.37a	0.95 ± 0.39a
(E)-2-Decenal	1,634	0.01 ± 0bc	0.02 ± 0ab	0.03 ± 0a	0.01 ± 0bc	0 ± 0c
Citral	1,717	0.76 ± 0.02a	0.12 ± 0.03a	0.64 ± 0.16a	0.27 ± 0.13a	1.01 ± 0.38a
Subtotal		14.41 ± 1.32ab	14.24 ± 3.66a	14.57 ± 3.28a	10.53 ± 2.31ab	6.73 ± 1.82b
**Alcohols**						
1-Butanol	1,138	0.48 ± 0.04a	0.3 ± 0.05a	0.52 ± 0.03a	0.3 ± 0.01a	0.65 ± 0.16a
1-Hexanol	1,034	1.45 ± 0.37b	3.95 ± 0.22ab	5.08 ± 0.15ab	6.53 ± 1.88a	2.58 ± 0.28ab
(E)-2-Hexenol	1,394	0.4 ± 0.09a	0.8 ± 0.15a	1.28 ± 0.24a	3.66 ± 1.98a	0.79 ± 0.16a
1-Octen-3-ol	1,420	0.32 ± 0.03a	0.51 ± 0.14a	0.33 ± 0.02a	0.49 ± 0.03a	0.26 ± 0.05a
1-Heptanol	1,454	0.06 ± 0.01b	0.23 ± 0.05a	0.15 ± 0ab	0.13 ± 0.03ab	0.05 ± 0.01b
2-Ethylhexanol	1,499	0.16 ± 0.02a	0.18 ± 0.01a	0.18 ± 0.03a	0.19 ± 0.01a	0.15 ± 0.01a
1-Octanol	1,548	0.92 ± 0.09c	3.45 ± 0.83ab	4.01 ± 0.37a	1.28 ± 0.35bc	1.27 ± 0.42bc
(E)-2-Octen-1-ol	1,620	0.14 ± 0.01ab	0.16 ± 0.03ab	0.21 ± 0.01a	0.14 ± 0ab	0.1 ± 0.02b
(Z)-5-Octen-1-ol	1,626	0.1 ± 0b	0.18 ± 0.02b	0.48 ± 0.01a	0.11 ± 0.04b	0.11 ± 0.03b
(E)-5-Decen-1-ol	1,767	1.48 ± 0.16b	5.22 ± 0.95ab	7.62 ± 0.88a	4.35 ± 1.41ab	2.03 ± 0.53b
Phenylethyl alcohol	1,902	0.22 ± 0.04c	4.89 ± 0.81abc	9.25 ± 1.02a	5.51 ± 2.12ab	0.34 ± 0.09bc
Cinnamyl alcohol	2,252	0.06 ± 0.02a	0.01 ± 0b	0.01 ± 0b	0 ± 0b	0 ± 0b
Subtotal		5.79 ± 0.74b	19.88 ± 3.63ab	29.11 ± 4.15a	22.69 ± 13.19ab	8.34 ± 1.86b
**Acids**						
Acetic acid	1,445	0.17 ± 0.02a	0.11 ± 0.01a	0.09 ± 0.03a	0.12 ± 0.04a	0.13 ± 0.08a
Hexanoic acid	1,840	2.6 ± 0.15a	2.31 ± 0.18a	2.92 ± 0.74a	4.15 ± 2.03a	2.3 ± 0.17a
Heptanoic acid	1,962	0.16 ± 0.01a	0.1 ± 0.03a	0.12 ± 0.03a	0.18 ± 0.11a	0.11 ± 0.01a
Octanoic acid	2,034	0.23 ± 0.04ab	0.18 ± 0.02ab	0.26 ± 0.02a	0.18 ± 0.02ab	0.15 ± 0.01b
Nonanoic acid	2,174	1.1 ± 0.12ab	0.93 ± 0.05ab	1.52 ± 0.14a	0.68 ± 0.34b	0.98 ± 0.06ab
Geranic acid	2,287	17.89 ± 1.32a	0.89 ± 0.2b	2.05 ± 0.09b	0.63 ± 0.16b	13.36 ± 3.14a
Subtotal		4.25 ± 0.51a	3.63 ± 0.39a	4.90 ± 1.27a	5.33 ± 4.20a	3.67 ± 0.10a
**Terpenes**						
D-Limonene	1,201	0.09 ± 0.01a	0.01 ± 0b	0.02 ± 0b	0.01 ± 0b	0.09 ± 0.02a
Linalool	1,553	1.04 ± 0.11a	0.03 ± 0.01b	0.14 ± 0.02b	0.03 ± 0b	1.08 ± 0.37a
Citronellol	1,755	1.17 ± 0.16a	0.03 ± 0.01c	0.14 ± 0.01bc	0.03 ± 0.01c	0.86 ± 0.33ab
Nerol	1,767	0.09 ± 0a	0.01 ± 0b	0.03 ± 0b	0 ± 0b	0.1 ± 0.03a
Geraniol	1,830	11.07 ± 3.16a	2.44 ± 0.41b	9.29 ± 0.49ab	1.96 ± 0.33b	9.55 ± 2.06ab
Subtotal		13.46 ± 5.80a	2.52 ± 0.73b	9.61 ± 0.88ab	2.03 ± 0.58b	11.68 ± 2.92a
**Ketones**						
2-Octanone	1,275	4.82 ± 0.02a	5.27 ± 0.13a	4.73 ± 0.75a	5.18 ± 0.14a	4.8 ± 0.1a
Acetophenone	1,645	0.1 ± 0.03a	0.06 ± 0ab	0.08 ± 0ab	0.04 ± 0ab	0.02 ± 0b
Subtotal		4.92 ± 0.02a	5.33 ± 0.24a	4.80 ± 1.30a	5.22 ± 0.25a	4.82 ± 0.18a
**Furan**						
2-Pentylfuran	1,229	0.08 ± 0c	0.11 ± 0bc	0.33 ± 0.02a	0.18 ± 0.02b	0.11 ± 0.03bc
Subtotal		0.08 ± 0c	0.11 ± 0bc	0.33 ± 0.02a	0.18 ± 0.02b	0.11 ± 0.03bc
Total		64.60 ± 5.90a	53.98 ± 4.87a	72.58 ± 7.49a	54.50 ± 20.2a	59.61 ± 1.34a

*Data are expressed as M ± SD from three technologic replicates. 1 abundance unit is equal to 100 μg/g fresh weight. Numbers in the same row followed by the same letter within each cultivar are not significantly different by Tukey's HSD test (95% confidence)*.

**Figure 2:**
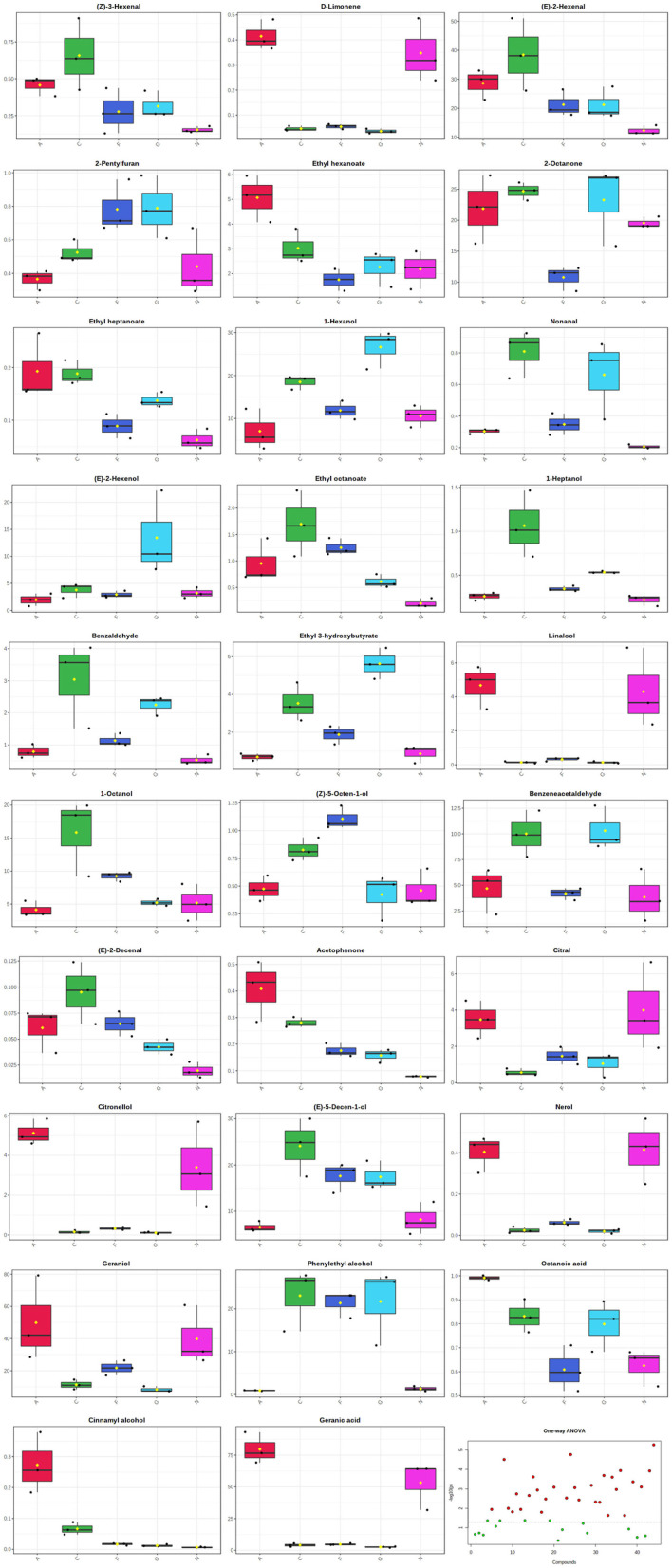
Box and whisker plots showing the significantly different volatiles identified in the five muscadine grape cultivars. Vertical bars represent the normalized relative concentration of volatiles, and the *x*-axis depicts the different muscadine grape cultivars. A, Alachua; C, Carlos; F, Fry; G, Granny Val; N, Noble.

The level of each VOC was evaluated using its relative area toward the internal standard. Geranic acid, geraniol, 2-octanone, and (*E*)-2-hexenal components were found to be with the greatest concentration values in the Alachua and Noble MG cultivars. The main components quantified in the Carlos, Fry, and Granny Val MG cultivars were (*E*)-5-decen-1-ol, phenylethyl alcohol, 1-hexanol, 2-octanone, and (*E*)-2-hexenal (Table 1). A log transformation was performed to allow the in-depth analysis of all the VOCs detected (Figure 2). One-way ANOVA combined with Tukey's HSD test (*P* < 0.05) was used to estimate the significant differences in VOC content among the five MG cultivars, resulting in 29 significantly different (*P* < 0.05) metabolites (Figure 2).

The cultivar (Ju et al., 2021), cultural practices (Golombek et al., 2021), and postharvest biological control (Mencarelli and Bellincontro, 2018) were the most influential factors affecting the volatile compositions and the production of grapes. In the present study, all vines were grown under the same conditions using the same horticultural practices. In addition, strictly identical extraction conditions and analytical parameters were used for all samples. The influence of the environmental factors and technical parameters on the composition and production of volatiles was negligible. Therefore, by bringing together these extensive factors, we demonstrated in our study that the main source of variance is closely related to the cultivar. The predominant compounds contributing to the MG volatile profiles agree with those found previously, among which the hexenal, (*E*)-2-hexenal, and 1-hexanol were reported as the major constituents of six clones of spine grape berries (Ju et al., 2021), and geraniol and ethyl acetate were reported as the main constituents of Cowart MG berries (Lee et al., 2016). The (*E*)-2-hexenal and 1-hexanol identified here have been reported as the dominant C_6_ volatile compounds in *V. vinifera* cultivars. However, there was a wide variation in the concentration and percentage of the C_6_ volatiles contributing to the total volatiles (Yang et al., 2009). This finding indicated that the composition and concentration of VOCs in MG cultivars could vary with the genetic background.

### Multivariate PCA and PLS-DA Analyses of the HS-SPME-GC-MS Data

**Figure 3:**
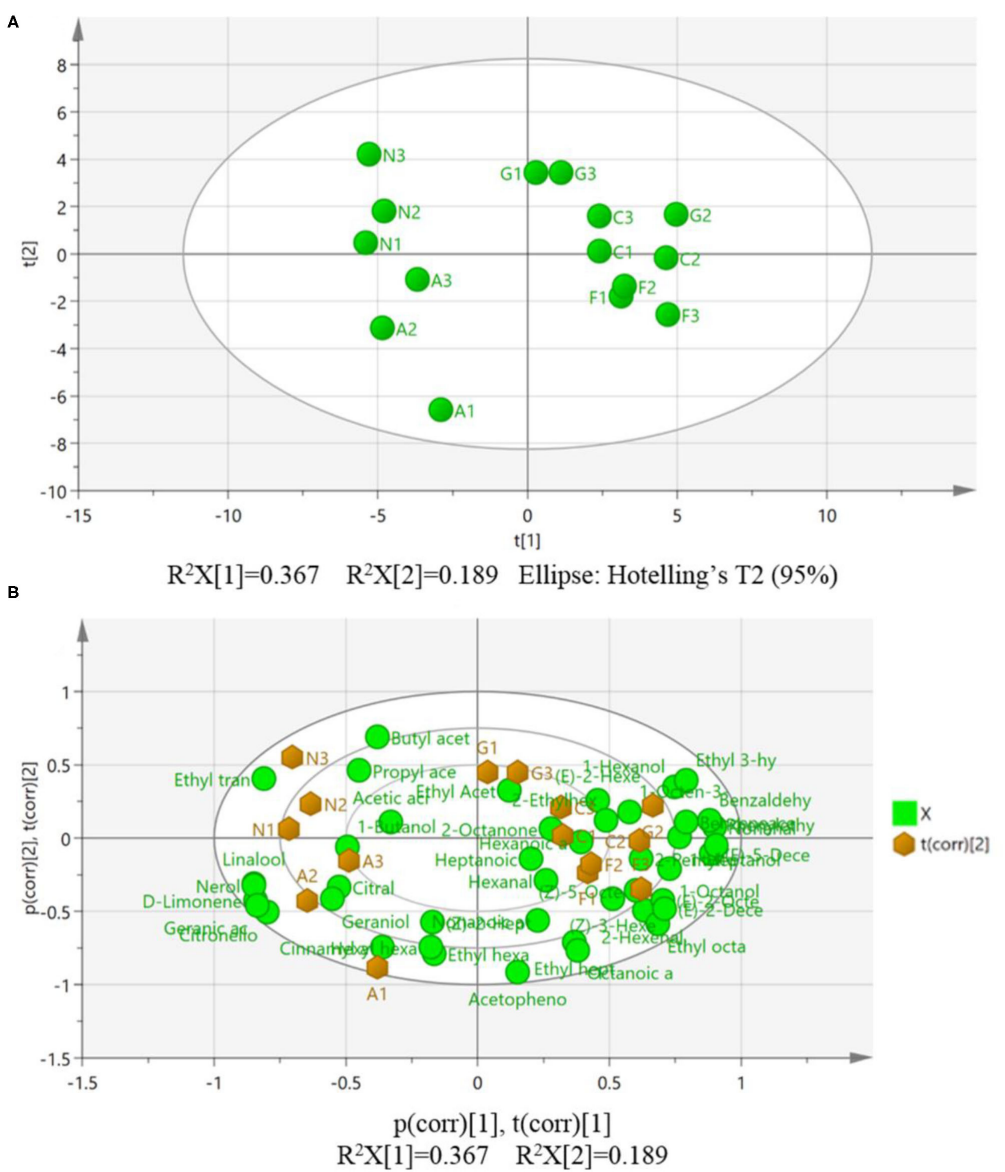
Principal component analysis (PCA) showing the main differences in the fruit volatiles of the five muscadine grape cultivars. **(A)** PCA score plot of all the investigated samples; **(B)** PCA biplot of the variables associated with 15 investigated samples. A, Alachua; C, Carlos; F, Fry; G, Granny Val; N, Noble; t[1], the first principal component score; t[2], the second principal component score.

Multivariate analysis of the dataset using PCA was primarily performed to visualize the overall differentiation and intrinsic variation of VOCs among the five MG cultivars (Figure 3A). PCA is an unsupervised chemometric method that reduces dimensionality and visualizes the main correlations and variability of a complex dataset (Vidal et al., 2016). In the PCA score scatter plot, the cultivars Carlos, Fry, and Granny Val were located at the right side of the score plot towards the center, while the distribution of samples from the center to the left side of the score plot comprised the cultivars Alachua and Noble. A clear differentiation among the five MG cultivars can be observed, in particular, the bronze-colored cultivars are distributed far away from the purple-black colored cultivars, as depicted in Figure 3A. All the investigated samples were located within the 95% confidence interval, which indicated that no outliers existed in the whole dataset. The accumulated variance contribution rate R^2^X reached 0.667, with the first and second principal components (PCs) carrying data variance of 36.7 and 18.9%, respectively. The Q^2^ value of the PCA model was 0.254 (Figure 3A).

Since in a PCA model, the directions in the score plot correspond to the directions in the loading plot, a comparison of these two plots can be used to identify which variables (loadings) have the greatest influence on the class separation of the different samples (scores) (Lubes and Goodarzi, 2017). Based on these criteria, the individual volatiles attributed to the variation in the five MG cultivars were graphically illustrated in a biplot (Figure 3B).

**Figure 4:**
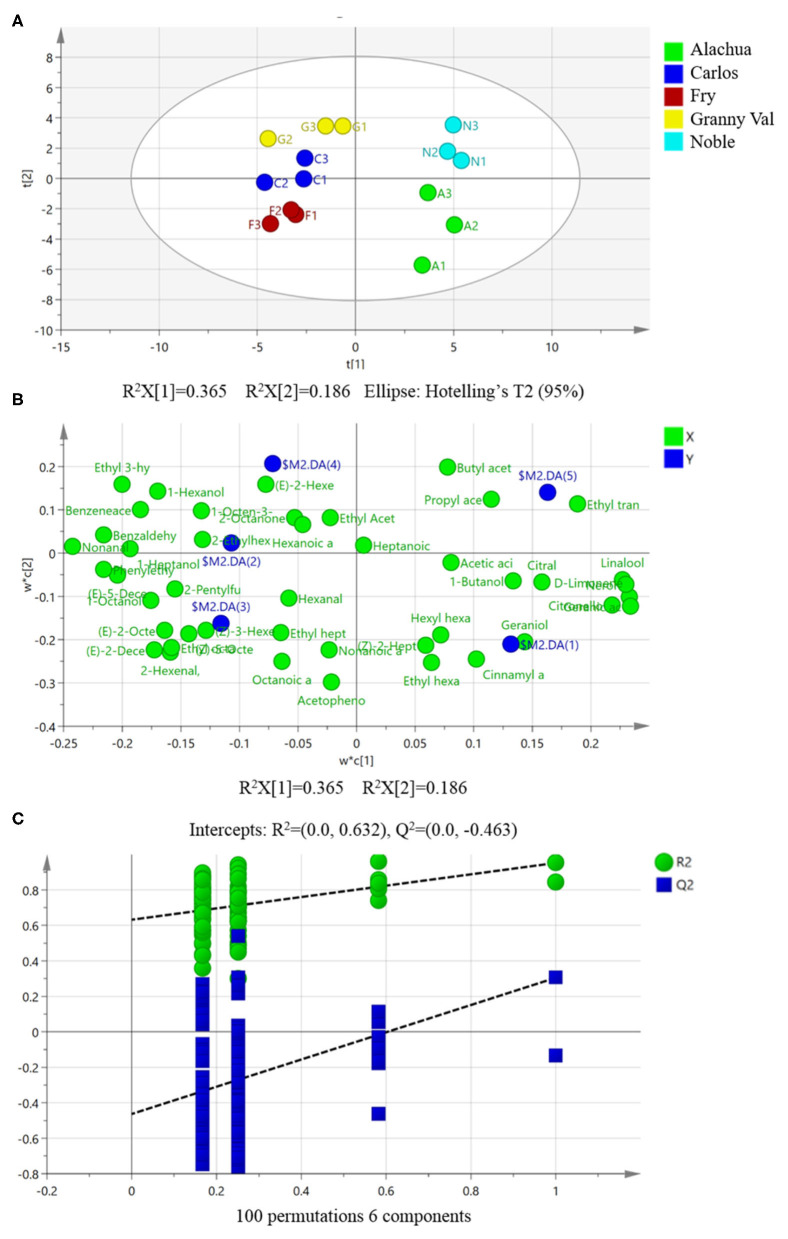
Partial least-squares discriminate analysis (PLS-DA) showing a better separation among the five muscadine grape cultivars based on the fruit volatiles. **(A)** PLS-DA score plot of all the investigated samples; **(B)** loadings plot of variables associated with different muscadine grape cultivars; **(C)** permutation test for PLS-DA model validation. A, Alachua; C, Carlos; F, Fry; G, Granny Val; N, Noble; t[1], the first principal component score; t[2], the second principal component score.

Partial least-squares discriminant analysis is a supervised multivariate statistical analysis and a variant of the PLS regression approach, which is widely used to construct a multidimensional model to predict features, discriminating between different samples, and further potential biomarkers explorations (Kalivodová et al., 2015). Therefore, PLS-DA was subsequently applied to perform an even better separation of the MG cultivars. Similarly, the score scatters plot of PLS-DA showed that the clusters of bronze-colored cultivars were located far from the purple black-color cultivars. Compared with the PCA model (Figure 3A), a noticeable improvement in the distinction of the five MG cultivars was observed (Figure 4A). All data points were within the 95% confidence interval (Figure 4A) and consistent with the results of the PCA model.

The PLS-DA model revealed that the corresponding values of R^2^X(cum), R^2^Y(cum), and Q^2^(cum) were 0.823, 0.927, and 0.503, respectively (Figure 4A). In the PLS-DA model, the R^2^X and R^2^Y values were utilized to describe the total explained variation in X and Y, respectively, and were represented by the PCs. At the same time, the Q^2^ parameter was used to assess the robustness of the model (Kalivodová et al., 2015). The R^2^Y and Q^2^ parameters of PLS-DA were significantly elevated (>0.5) (Figure 4A), indicating a valid and robust model (Kalivodová et al., 2015).

In this study, the PLS-DA model was shown to be a tool that could be used to investigate whether the five MG cultivars can be effectively discriminated (Figure 4A) and enable the visualization of the volatiles that contributed the most to the corresponding discrimination of the five MG cultivars (Figure 4B). The loadings of the variables on the two PLS-DA components are graphically illustrated in Figure 4B. More specifically, geraniol and cinnamyl alcohol were demonstrated to be essential for characterizing the Alachua MG cultivar in the loading plot of the PLS-DA. At the same time, ethyl trans-2-butenoate and propyl acetate were essential compounds to characterize the Noble MG cultivar. Furthermore, 2-Ethyl-1-hexanol, (*Z*)-3-hexenal, and (*E*)-2-hexenol were closely related to the Carlos, Fry, and Granny Val cultivars, respectively (Figure 4B).

The study further preformed 100 permutation tests to validate the PLS-DA model and are summarized in Figure 4C. The permutated models resulted in all R^2^ (representing the explained variance) and Q^2^ (representing the predictive capability) values (*Y*-axis data) on the left being lower than the original points on the right, indicating that the original model is statistically viable (Figure 4C).

### Specific Volatile Markers of the Five MG Cultivars

The potential markers to distinguish different samples can be selected using the PLS-DA model (Lubes and Goodarzi, 2017). The VIP scores in the PLS-DA model estimate the importance of each *x* variable for each *x* variate in the prediction model and summarize the contribution that a variable makes to the model (Chong and Jun, 2005). Generally, a VIP score greater than 1 is considered a criterion for variable selection. A VIP score lower than 0.5 indicates that the variable is unimportant for the model classification and discrimination (Chong and Jun, 2005). In this study, by setting a threshold value of 1 for the VIP score in the PLS-DA model, 24 volatile metabolites were identified as crucial differential volatiles (Table 2). Combining the VIP scores in Table 2 and the ANOVA results in Figure 2, five volatile markers that discriminate the five MG cultivars, namely 2-pentylfuran, 1-heptanol, ethyl hexanoate, (*Z*)-3-hexenal, and phenylethyl alcohol, were obtained.

**Table 2:** The variable importance in projection (VIP) scores within the partial least-squares discriminate analysis (PLS-DA) model.

**Variable ID (Primary)**	**Variable importance in projection (VIP) scores**
2-Pentylfuran	1.41
(Z)-5-Octen-1-ol	1.28
Cinnamyl alcohol	1.24
1-Heptanol	1.18
1-Octanol	1.17
(E)-2-Hexenol	1.16
Ethyl 3-hydroxybutyrate	1.14
Nonanoic acid	1.12
Citral	1.11
(Z)-2-Heptenal	1.09
Octanoic acid	1.08
Acetophenone	1.07
Hexanoic acid	1.03
Geraniol	1.03
Ethyl trans-2-butenoate	1.03
1-Butanol	1.02
(E)-2-Decenal	1.01
1-Hexanol	1.01
(E)-2-Hexenal	0.99
Ethyl hexanoate	0.99
(Z)-3-Hexenal	0.99
Nerol	0.97
Heptanoic acid	0.97
Phenylethyl alcohol	0.95
Ethyl octanoate	0.94
1-Octen-3-ol	0.94
Hexanal	0.94
D-Limonene	0.93
Ethyl Acetate	0.93
(E)-5-Decen-1-ol	0.93
Geranic acid	0.92
Citronellol	0.91
Linalool	0.90
Benzeneacetaldehyde	0.89
Ethyl heptanoate	0.89
Hexyl hexanoate	0.89
(E)-2-Octen-1-ol	0.88
Nonanal	0.88
Butyl acetate	0.88
Propyl acetate	0.85
Benzaldehyde	0.85
2-Octanone	0.82
2-Ethylhexanol	0.70
Acetic acid	0.66

**Figure 5:**
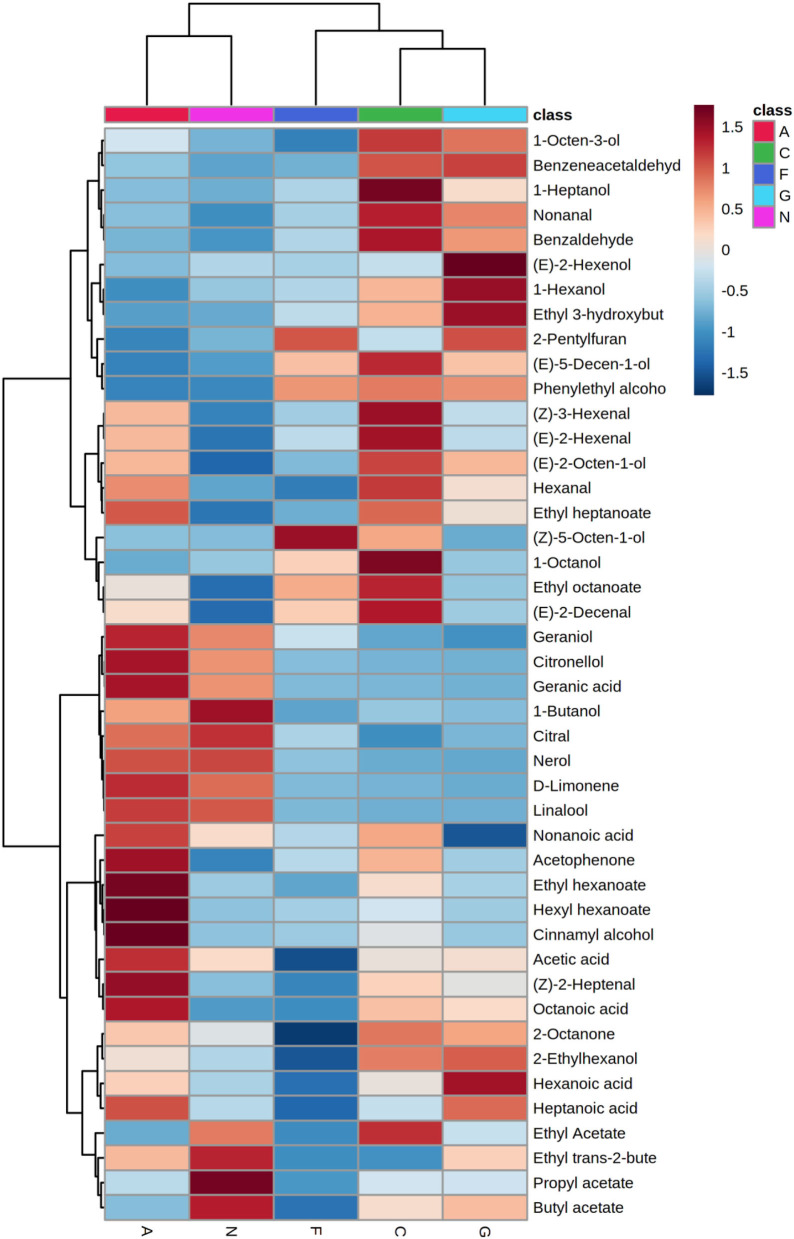
Hierarchical clustering heatmap of the different volatiles in the five muscadine grape cultivars. Each colored cell on the map corresponds to a relative concentration of a volatile, with the samples in the rows and compounds in the columns. A, Alachua; C, Carlos; F, Fry; G, Granny Val; N, Noble.

Subsequently, a hierarchical cluster analysis (HCA) dendrogram was constructed to visualize the content differences of the potentially characterized VOCs (Figure 5). The HCA results revealed that the key volatiles were grouped into two classes. The first class comprised nine volatiles more abundant volatiles in the Carlos, Fry, and Granny Val MG cultivars. The second contained 15 volatiles correlated with the Alachua and Noble MG cultivars. This result was in accordance with the above multivariate analysis results.

Fruit aroma is highly influential on the overall flavor and consumer preference (Lin et al., 2019). As an aroma-dense fruit, the MG grape is a promising cultivar for future breeding efforts to attain the desired volatile aroma of table grapes and resulting wine. The characterization of the volatile profiles of the aroma-dense MG cultivars is the first step in elucidating the possible molecular mechanisms underlying volatile synthesis and the future genetic improvement of grape aroma. In this study, the volatile profiles of five newly introduced MG cultivars were comprehensively investigated and evaluated. The results opened the avenues to attain a cultivar with the desired flavor in the future.

## Conclusions

Muscadine grape is an aroma-dense fruit and has been increasingly appreciated by growers, breeders, and consumers worldwide. Recently, five MG cultivars, namely Alachua, Carlos, Fry, Granny Val, and Noble, have been successfully introduced and cultivated in southern China. In the current study, the volatile constituents of the berries of the five MG cultivars were isolated using HS-SPME and analyzed using GC-MS. The results identified 44 different compounds in the studied cultivars by comparing the mass spectra and retention index with authentic standards, NIST libraries, and literature data. These volatiles were divided into esters, aldehydes, alcohols, fatty acids, terpenes, ketones, and furan based on their chemical nature. ANOVA, combined with Tukey's HSD test, revealed that the significant differences among the five cultivars are due to the quantitative differences of the 29 volatiles. Multivariate PCA and PLS-DA analyses showed a clear differentiation among the five cultivars, particularly the bronze-colored cultivars and the purple-black colored cultivars. The volatiles that contribute the most to the corresponding discrimination of five cultivars were highlighted. The implications of these findings were also discussed. This investigation is the first in-depth exploration of the volatile profiles of the aroma-dense MG.

## Data Availability Statement

The original contributions presented in the study are included in the article/Supplementary Material, further inquiries can be directed to the corresponding author/s.

## Author Contributions

HD, QD, and HX conceived and designed the research and checked and revised the manuscript. HD, RH, ML, YL, and YZ performed the experiments. HD, LL, DL, XZ, ML, and XL analyzed the data. HD prepared and wrote the manuscript. All authors contributed to this article and approved the submitted version.

## Funding

This study was financially supported by the Sichuan Science and Technology Programs (2020YFH0144 and 2020JDKP0039) and Sichuan Science Technology Resource Sharing Service Platform (Grape Resources).

## Conflict of Interest

The authors declare that the research was conducted in the absence of any commercial or financial relationships that could be construed as a potential conflict of interest.

## Publisher's Note

All claims expressed in this article are solely those of the authors and do not necessarily represent those of their affiliated organizations, or those of the publisher, the editors and the reviewers. Any product that may be evaluated in this article, or claim that may be made by its manufacturer, is not guaranteed or endorsed by the publisher.
