# Supplementary figures and images for: Comparison of the Fruit Volatile Profiles of Five Muscadine Grape Cultivars (Vitis rotundifolia Michx.) Using HS-SPME-GC/MS Combined With Multivariate Statistical Analysis
Source: Front Plant Sci. 2021 Oct 25;12:728891. doi: 10.3389/fpls.2021.728891 (PMC8572961; doi:10.3389/fpls.2021.728891)

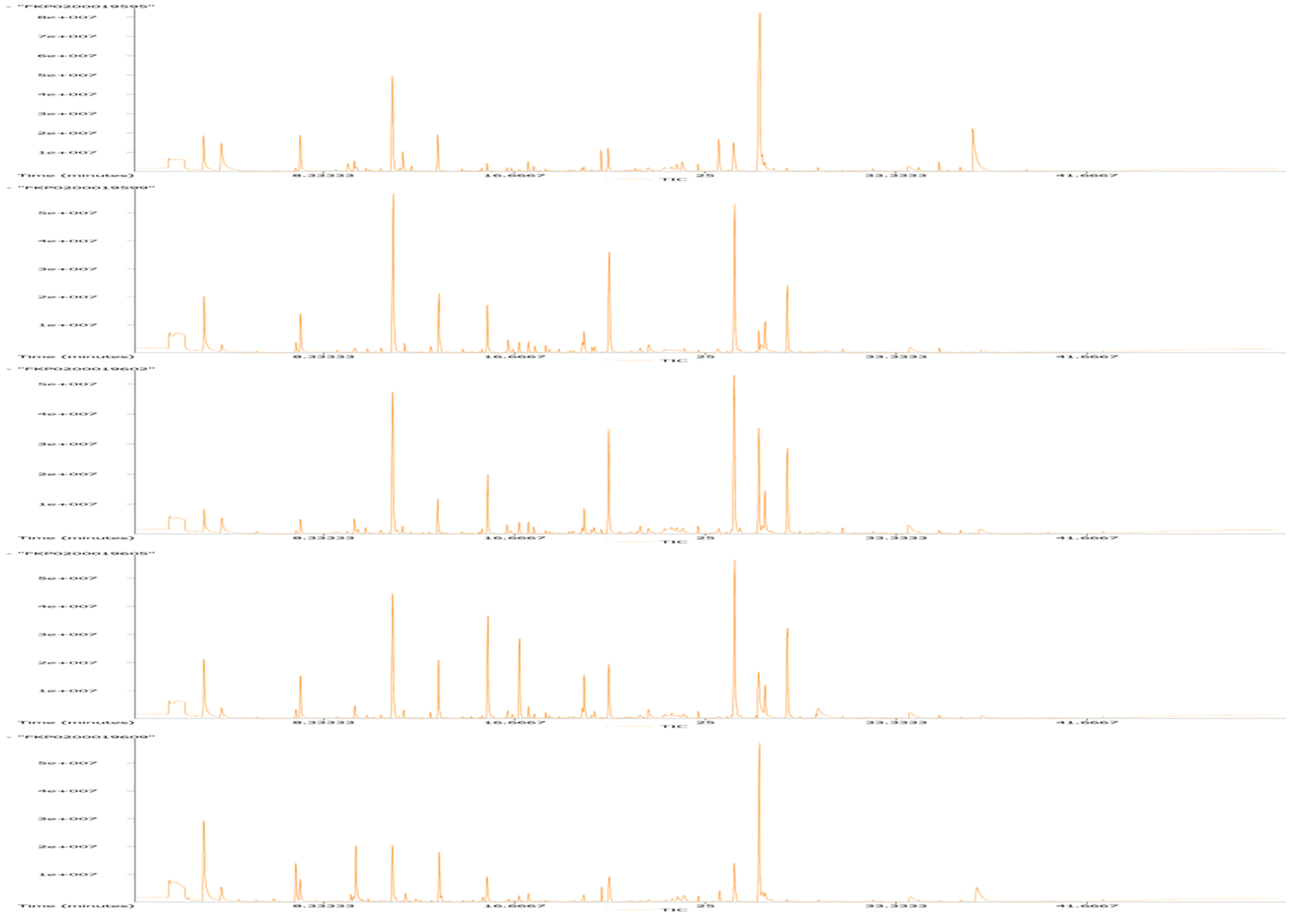

Supplement: Supplementary file 1 [file Image_1.TIF]
